# Formation of two-dimensional MoS_2_ and one-dimensional MoO_2_ nanowire hybrids

**DOI:** 10.1186/s42649-019-0020-6

**Published:** 2019-12-17

**Authors:** Aram Yoon, Zonghoon Lee

**Affiliations:** 1 School of Materials Science and Engineering, Ulsan National Institute of Science and Technology (UNIST), Ulsan, 44919 Republic of Korea; 2 Center for Multidimensional Carbon Materials, Institute for Basic Science (IBS), Ulsan, 44919 Republic of Korea

**Keywords:** 2D material, Oxidation, MoS_2_, MoO_2_, Nanowire

## Abstract

Oxidation of two-dimensional (2D) transition metal dichalcogenides have received great interests because it significantly influences their electrical, optical, and catalytic properties. Monoclinic MoO_2_ nanowires grow along the zigzag direction of 2D MoS_2_ via thermal annealing at a high temperature with a low oxygen partial pressure. The hybrids of semiconducting 2D MoS_2_ and metallic 1D MoO_2_ nanowires have potential to be applied to various devices such as electrical devices, gas sensors, photodetectors, and catalysts.

## Description

Two-dimensional (2D) transition metal dichalcogenides (TMDs) have received great interests because of their outstanding electrical, optical, and catalytic properties. However, these properties are significantly influenced by oxidation. Moreover, heterostructure of transition metal oxides (TMOs) and TMDs are emerging as promising candidates for functional devices such as gas sensor, photodetector, and catalyst. Therefore, synthesizing multidimensional TMDs and TMOs hybrids are actively studied. (Molina-Mendoza et al., 2016).

Herein, we propose a new synthetic route for growth of 1D MoO_2_ nanowires and 2D MoS_2_ flakes hybrids via thermal oxidation of MoS_2_. Oxidation into MoO_3_ is common phenomenon in ambient conditions, but metallic MoO_2_ nanowires grow at a high temperature with a low oxygen partial pressure. The hybrids are investigated using transmission electron microscopy (TEM) and scanning electron microscopy (SEM). TEM imaging was performed with a FEI Titan Cube G2 60–300 instrument operated at 200 kV with an image aberration corrector, and SEM imaging was performed with Hitachi High-Technologies S-4800.

**Fig. 1:**
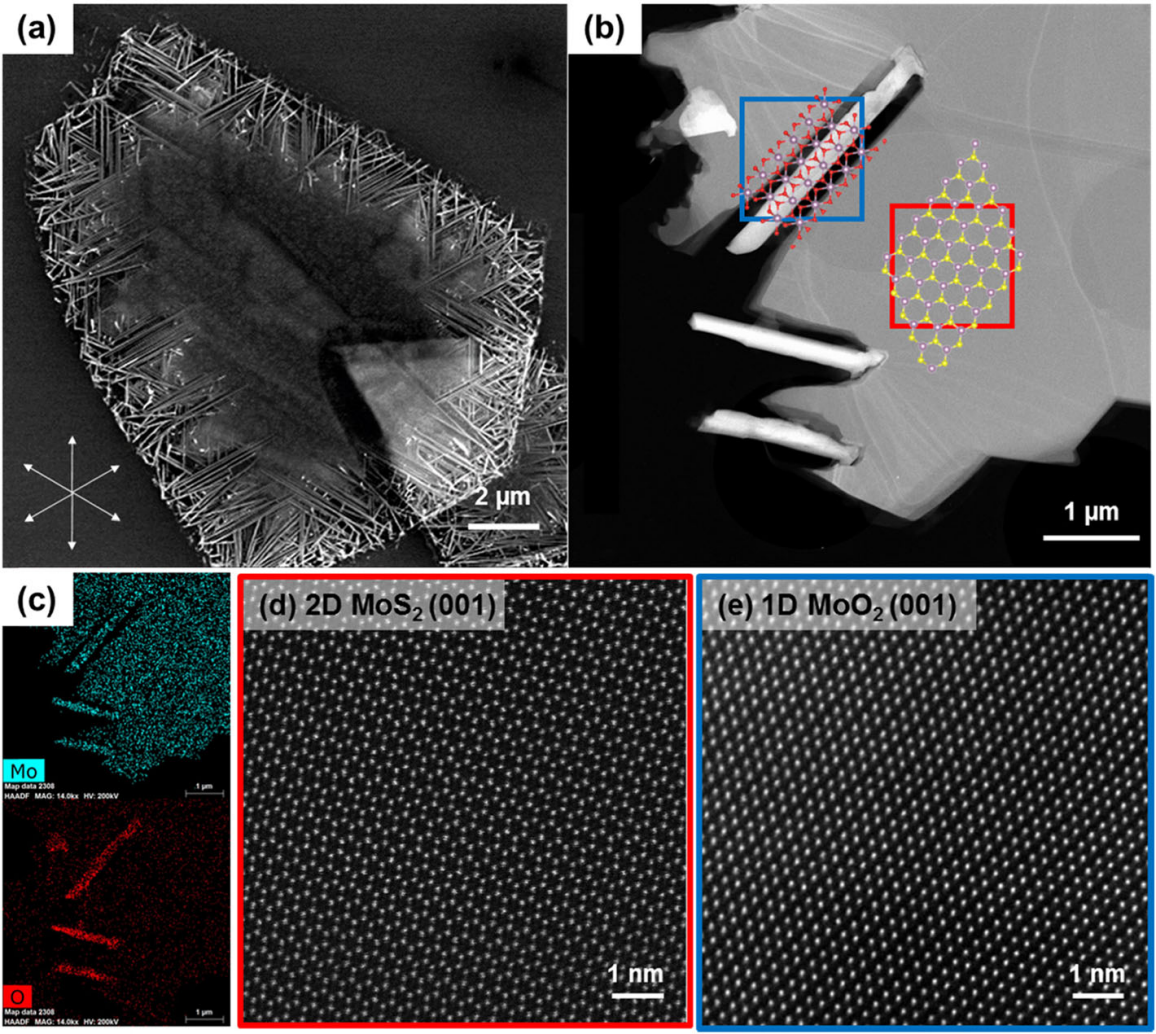
(**a**) SEM, (**b**) STEM, and corresponding (**c**) EDS images of 1D MoO_2_ nanowires and 2D MoS_2_ hybrids. Atomic-scale STEM images (**d**) MoS_2_ and (**e**) MoO_2_ were taken from the marked position as a red box and blue box in (**b**), respectively

In Fig. 1a, SEM image clearly shows that 1D nanowires are grown along the edges of 2D MoS_2_ flake, and the energy dispersive spectroscopy results in Fig. 1c confirm that they are molybdenum oxides. Moreover, Fig. 1d-e atomic-scale scanning TEM (STEM) images show that the molybdenum oxides are monoclinic MoO_2_ with distorted rutile structure. The growth direction of nanowires is [100] which is same as the results in previous papers. (Vogl et al., 2019) In addition, MoO_2_ nanowires are dominantly grown in six radial orientations at MoS_2_ as indicated by the arrows in Fig. 1a. These orientations are consistent with the zigzag directions of MoS_2_.

High contact resistance of TMDs is one of the key bottlenecks for applying TMDs to electrical devices. However, synthesizing the hybrids of semiconducting 2D MoS_2_ and metallic 1D MoO_2_ nanowires can be a solution. Metallic MoO_2_ nanowires can be adopted as an electrode of MoS_2_ directly, and hybridization between MoO_2_ and MoS_2_ might remove the Schottky barrier height and decrease the contact resistance. This simple synthetic route can be applied to many other 2D materials, and these 1D oxides-2D material hybrids can be used for not only electronic devices, but also catalytic, and optical devices.

## Data Availability

Not applicable.
